# Special Issue “Mechanisms and Novel Therapeutic Approaches for Neurodegenerative Diseases”

**DOI:** 10.3390/biomedicines12112554

**Published:** 2024-11-08

**Authors:** Fernando Cardona

**Affiliations:** 1 Unitat de Genètica Molecular, Instituto de Biomedicina de Valencia, Consejo Superior de Investigaciones Científicas (CSIC), 46010 Valencia, Spain; fcardona@ibv.csic.es; 2 Centro de Investigación Biomédica en Red en Enfermedades Neurodegenerativas (CIBERNED), Instituto de Salud Carlos III (CIBER-ISCIII), 28029 Madrid, Spain; 3 Departamento de Biotecnología, ETSIAMN, Universitat Politècnica de València, 46022 Valencia, Spain

Neurodegenerative diseases, including Alzheimer’s disease (AD), Parkinson’s disease (PD), and amyotrophic lateral sclerosis (ALS), are among the major health problems of the elderly, and represent a major global health challenge due to their increasing prevalence and complex pathophysiological mechanisms. These pathologies cause problems of autonomy for the affected individuals, as well as major public health problems and significant costs for healthcare systems [1].

The mechanisms underlying neurodegeneration are sometimes common to different diseases, and in other cases, they are differentiated. In some cases, the mechanisms are unknown, and in others, although the mechanisms are known, there are no effective therapies to treat these diseases. In fact, there are few therapies to treat these diseases to prevent, slow, or stop neurodegeneration. Understanding these mechanisms is crucial for the development of innovative therapeutic strategies. This Editorial discusses the underlying mechanisms of these diseases, highlights the role of model systems, and explores emerging therapeutic approaches that hold promise for future treatment paradigms.

The pathophysiology of neurodegenerative diseases is complex and often involves the interaction of genetic, environmental, and biochemical factors. In AD, the accumulation of amyloid beta plaques and tau protein tangles disrupts synaptic function and triggers neuroinflammation, ultimately leading to neuronal death [2]. In PD, the progressive loss of dopaminergic neurons in the substantia nigra is primarily associated with the accumulation of α-synuclein aggregates, which contribute to cellular toxicity [3]. ALS is characterized by motor neuron degeneration with pathological features including the mislocalization or misfunction of SOD1, TDP-43, FUS, and C9orf72, and mitochondrial dysfunction that precedes motor neuron degeneration [4]. Understanding these mechanisms is essential to identify potential therapeutic targets. For example, the role of neuroinflammation in AD has led to research into anti-inflammatory agents, while targeting α-synuclein aggregation is a key focus for PD therapies. An example of a gene therapy proposal to reduce α-synuclein aggregation is included in this Special Issue [5], targeting the interaction of α-synuclein with UBL3 by silencing MGST3.

The study of neurodegenerative diseases has benefited greatly from using different model systems. Animal models, such as transgenic mice expressing human mutations associated with AD or PD, provide valuable insights into disease progression and the efficacy of potential treatments. These models allow researchers to observe the effects of genetic modifications and therapeutic interventions in vivo. Altay and colleagues [6] propose using NAD+ precursors to improve metabolic functions in animal models of neurodegenerative diseases.

Cellular models, including induced pluripotent stem cells (iPSCs), have also gained traction. iPSCs can be differentiated into relevant cell types, such as neurons and glial cells, providing a platform to study disease mechanisms at the cellular level. These systems enable high-throughput drug screening and testing of novel compounds that may modify disease pathways. The blood–brain barrier (BBB) plays a crucial yet paradoxical role in neurodegenerative diseases. While it acts as a protective shield, regulating the entry of harmful substances and pathogens into the brain, its dysfunction can exacerbate diseases such as AD and PD. Increased permeability allows toxins to accumulate and trigger neuroinflammation, creating a damaging cycle that accelerates neuronal loss. The BBB also complicates treatment strategies, as many therapeutic agents struggle to penetrate this barrier effectively. As we advance our understanding of the complex role of the BBB in neurodegeneration, it is essential to explore innovative approaches that not only restore its integrity but also facilitate the targeted delivery of treatments. Overcoming these challenges could pave the way for more effective interventions and improved outcomes for those affected by these debilitating diseases.

Recent advances in therapeutic strategies for neurodegenerative diseases have broadened the landscape of treatment options. Disease-modifying therapies, which aim to halt or slow disease progression rather than alleviate symptoms, have emerged as a key focus. For PD, gene therapy approaches are being investigated, including using adeno-associated viruses for the delivery of genes encoding neuroprotective factors or enzymes that increase dopamine synthesis. In addition, small molecules that inhibit α-synuclein aggregation or enhance its clearance are being developed. Moreover, using antisense oligonucleotides (ASOs) targeting specific gene mutations has shown promise in preclinical and early clinical trials. These ASOs can reduce the expression of toxic proteins associated with the disease, potentially altering its course [7].

Understanding the mechanisms underlying neurodegenerative diseases is essential for the development of effective therapies. With advances in model systems and innovative therapeutic approaches, there is hope for more targeted and effective treatments. Continued research into the complex interplay of genetic, cellular, and environmental factors will be critical to overcoming the challenges posed by these debilitating diseases. As we move forward, a multidisciplinary approach integrating genetics, pharmacology, and neurology will be essential to unlock the potential for meaningful therapeutic interventions in neurodegenerative diseases.

## References

[1] WHO (2020). Global Action Plan on the Public Health Response to Dementia 2017–2025 (2017).

[2] Wilson D.M., Cookson M.R., Van Den Bosch L., Zetterberg H., Holtzman D.M., Dewachter I. (2023). Hallmarks of neurodegenerative diseases. Cell.

[3] Baggett D., Olson A., Parmar M.S. (2024). Novel approaches targeting α-Synuclein for Parkinson’s Disease: Current progress and future directions for the disease-modifying therapies. Brain Disord..

[4] Riva N., Domi T., Pozzi L., Lunetta C., Schito P., Spinelli E.G., Cabras S., Matteoni E., Consonni M., Bella E.D. (2024). Update on recent advances in amyotrophic lateral sclerosis. J. Neurol..

[5] Yan J., Zhang H., Tomochika Y., Chen B., Ping Y., Islam M.S., Aramaki S., Sato T., Nagashima Y., Nakamura T. (2023). UBL3 Interaction with α-Synuclein Is Downregulated by Silencing MGST3. Biomedicines.

[6] Altay O., Yang H., Yildirim S., Bayram C., Bolat I., Oner S., Tozlu O.O., Arslan M.E., Hacimuftuoglu A., Shoaie S. (2024). Combined Metabolic Activators with Different NAD+ Precursors Improve Metabolic Functions in the Animal Models of Neurodegenerative Diseases. Biomedicines.

[7] Anwarkhan S., Koilpillai J., Narayanasamy D. (2024). Utilizing Multifaceted Approaches to Target Drug Delivery in the Brain: From Nanoparticles to Biological Therapies. Cureus.

